# Supplementary material for: Polyketide synthase gene clusters in the ericoid mycorrhizal fungus Oidiodendron maius: genome-wide prediction, expression profiling, and functional characterization of knockout mutants
Source: Front Plant Sci. 2026 Sep 16;17:1924430. doi: 10.3389/fpls.2026.1924430 (PMC13623903; doi:10.3389/fpls.2026.1924430)
Supplement: Supplementary file 1 [file SupplementaryFile1.zip › Supplementary Materials/Supplementary Folder 1.zip!/Oidma1_AssemblyScaffolds_Repeatmasked/knownclusterblast/cluster36/jgi.p_Oidma1_131674_mibig_hits.html]

| MIBiG Protein | Description | MIBiG Cluster | MiBiG Product | % ID | % Coverage | BLAST Score | E-value |
| --- | --- | --- | --- | --- | --- | --- | --- |
| EWG54267.1 | hypothetical\_protein | BGC0001190 | Fusaric\_acid\_biosynthetic\_gene\_cluster | 32 | 96.5587044534 | 222 | 8.9e-58 |
| AAP03116.1 | putative\_carboxyesterase | BGC0000874 | Blasticidin\_biosynthetic\_gene\_cluster | 43 | 35.6275303644 | 131 | 3.5e-30 |
| ABI94378.1 | TMC\_biosynthetic\_enzyme\_L1 | BGC0000157 | Tautomycetin\_biosynthetic\_gene\_cluster | 35 | 39.6761133603 | 111 | 3.8e-24 |
| ABV91296.1 | putative\_type\_B\_carboxylesterase | BGC0000158 | Tautomycetin\_biosynthetic\_gene\_cluster | 35 | 39.6761133603 | 111 | 3.8e-24 |
| ABW96543.1 | putative\_type\_B\_carboxylesterase | BGC0000159 | Tautomycin\_biosynthetic\_gene\_cluster | 32 | 40.4858299595 | 108 | 1.9e-23 |
| ADC96667.1 | carboxyesterase | BGC0001076 | Liposidomycin\_biosynthetic\_gene\_cluster | 33 | 41.7004048583 | 102 | 1e-21 |
| BAJ05894.1 | putative\_acyltransferase | BGC0000872 | A-90289\_biosynthetic\_gene\_cluster | 32 | 41.0931174089 | 99 | 1.5e-20 |
| ACQ63629.1 | acyltransferase | BGC0000875 | Caprazamycin\_biosynthetic\_gene\_cluster | 32 | 43.5222672065 | 98 | 2.5e-20 |
| AJO72733.1 | Type\_B\_carboxylesterase/acyltransferase | BGC0001381 | Brasilinolide\_biosynthetic\_gene\_cluster | 30 | 40.0809716599 | 95 | 1.6e-19 |
| AIG62138.1 | carboxylesterase\_family\_protein | BGC0000120 | Patulin\_biosynthetic\_gene\_cluster | 29 | 50.4048582996 | 91 | 3.1e-18 |
